# Detection of HIV-1 Resistance Mutations to Antiretroviral Therapy and Cell Tropism in Russian Patients Using Next-Generation Sequencing

**DOI:** 10.3390/pathogens15020144

**Published:** 2026-01-28

**Authors:** Artem Fadeev, Veronika Eder, Maria Pisareva, Valery Tsvetkov, Alexey Masharskiy, Kseniya Komissarova, Anna Ivanova, Nikita Yolshin, Andrey Komissarov, Alexey Mazus, Dmitry Lioznov

**Affiliations:** 1 Smorodintsev Research Institute of Influenza, Prof. Popova Str., 15/17, 197376 Saint Petersburg, Russia; afadeew@gmail.com (A.F.); creatacrea@yahoo.com (V.E.); pisarevamm@gmail.com (M.P.); suppcolor@gmail.com (V.T.); masharsky@gmail.com (A.M.); kseniya.sintsova1994@gmail.com (K.K.); nikita.yolshin@gmail.com (N.Y.); dlioznov@yandex.ru (D.L.); 2 Moscow City Center for AIDS Prevention and Control, 8th Sokolina Gora Str., 15, 105275 Moscow, Russia; mazusai@zdrav.mos.ru

**Keywords:** human immunodeficiency virus, antiretroviral therapy, drug resistance mutations, next generation sequencing

## Abstract

The use of antiretroviral therapy (ART) as the only effective way to control human immunodeficiency virus (HIV) infection results in HIV drug resistance. Next-generation sequencing (NGS) has become a common method for identifying drug-resistant variants and reducing analysis costs. The aim of this study was to develop an NGS-based protocol for identifying resistance mutations and cell tropism of HIV-1 in adult patients with and without treatment experience in Russia in 2024–2025. Plasma samples from adult HIV-infected patients from Russia were analyzed. Consensus nucleotide sequences of *pol* and *env* genes were obtained using NGS. HIV-1 drug resistance analysis was conducted using the Stanford University HIVdb database. CXCR4 cell tropism was predicted using an empirical rule classifier. A protocol for NGS of HIV-1 *pol* and *env* genes was developed. The most common HIV-1 surveillance mutations were in the reverse transcriptase. High levels of resistance were observed to non-nucleoside reverse transcriptase inhibitors (NNRTIs) and nucleoside reverse transcriptase inhibitors (NRTIs) in treatment-experienced patients and to NNRTIs in treatment-naïve patients. Low levels of resistance were observed to protease and integrase strand transfer inhibitors (INSTIs). CXCR4 cell tropism was extremely rare. NGS allows for the simultaneous processing of large data sets during epidemiological studies. The introduction of NGS-based protocols allows for performing ART efficiency and tropism monitoring at scale.

## 1. Introduction

Currently, the only effective way to control human immunodeficiency virus (HIV) infection is the simultaneous use of several drugs in an antiretroviral therapy (ART) regimen. The introduction of ART into clinical practice has increased the life expectancy and improved the quality of life of HIV-infected patients, as well as reducing the risk of infection transmission and the rate of its spread [1,2]. In Russia, ART has been widely used since 2006 within the framework of the national healthcare program; the proportion of patients receiving etiotropic therapy increased from 1% in 2005 to 35.5% in 2017 and amounted to 90% of those registered with dispensaries in 2024 [3]. Until 2020, the primary first-line ART regimens included two nucleoside reverse transcriptase inhibitors (NRTIs)—tenofovir (TDF) and zidovudine (AZT)—in combination with lamivudine (3TC) or emtricitabine (FTC), and one non-nucleoside reverse transcriptase inhibitor (NNRTI), primarily efavirenz (EFV). Recently, first-line ART regimens have also included a combination of two NRTIs and one integrase strand transfer inhibitor (INSTI), primarily dolutegravir (DTG). Second-line and subsequent ART regimens, however, include both INSTIs and boosted with ritonavir protease inhibitors (PIs) (atazanavir (ATV), darunavir (DRV), and lopinavir (LPV)) [4].

The widespread use of antiretroviral drugs and suboptimal adherence are contributing to the development and spread of HIV drug resistance (DR) due to the virus’s natural variability [5,6]. Among patients without ART experience, DR to at least one antiretroviral drug is detected in 12.7% of cases [7]. The average detection rate of drug resistance mutations (DRMs) in Russian patients with ART failure reaches 60–80% of all samples tested [8,9,10].

Mutations of HIV drug resistance to all ART drugs used in clinical practice are now known [11]. Cross-resistance can also develop between drugs within the same class [12]. Once acquired, DR can be transmitted from patient to patient and observed in HIV-infected patients with no prior treatment experience [13].

The biological characteristics of HIV play a significant role in the formation and persistence of DRMs [14]. Non-polymorphic mutations are known to frequently reduce HIV fitness. In the presence of antiretroviral drugs, viruses with such mutations have an evolutionary advantage; however, discontinuation of treatment is often accompanied by their rapid disappearance and the return of the wild-type virus with higher replicative activity [15]. A similar situation is often observed when a person is infected with a DR variant of HIV, where the original variant is replaced by the wild-type virus over the course of several months or years. Meanwhile, proviral DNA of variants with DRMs can be identified for quite a long time in latent T cells [16]. It is impossible to detect the presence of such mutations by analyzing HIV RNA in blood plasma [17]. After discontinuation of the ART to which HIV has developed DR, a reservoir of resistant variants of the pathogen remains in the human body for a long time [18]. There is evidence that the proportion of mutations in proviral DNA in therapy-naïve patients is significantly higher than that detected with standard RNA genotyping, and that these mutations persist for at least 1 year, irrespective of drug therapy [19].

Accessible, sensitive, and scalable technologies are needed to monitor the success of ART in HIV-1 infection. Currently used HIV-1 genotyping technologies are based on capillary sequencing. While these methods remain the gold standard for detecting clinically significant HIV DRMs, they are limited by high sequencing costs and low throughput. Capillary sequencing cannot detect mutations that are less abundant in the viral pool, also known as low-abundance DR variants [20]. Whole-genome next-generation sequencing (NGS) is becoming an increasingly common method for identifying low-abundance DR variants and reducing sample costs through sample pooling and massive parallel sequencing [20,21,22,23]. NGS is more sensitive because it can detect low-abundance DR variants and enables quantitative detection of DRMs in HIV [24]. As the number of new drugs for treating HIV-1 patients and widespread resistance increase, it is necessary to develop methods for genotyping HIV-1 with DR to detect resistance to new classes of drugs, such as capsid inhibitors, entry inhibitors, reverse transcriptase inhibitors, nucleoside analogues, and Rev inhibitors. To determine mutations of resistance of HIV-1 to antiretroviral drugs, test systems based on the amplification of fragments of the HIV genome are mainly used. In laboratory practice, approaches are used that combine the amplification of fragments of the HIV genome with subsequent sequencing of the amplification products by the Illumina method [21]. One of the few test systems registered for determining mutations of resistance of HIV-1 to antiretroviral drugs using the NGS method is the Sentosa SQ HIV Genotyping Assay manufactured by Vela Diagnostics (USA). This system is based on amplification of two regions of the HIV genome encoding the *pro* and *int* genes, as well as partially the *rev* gene. The resulting amplicons are then fragmented and used for library preparation and sequencing using Ion Torrent technology [25,26].

The aim of this study was to develop an NGS-based protocol for identifying resistance mutations and cell tropism of HIV-1, and to test the developed protocol on a set of samples collected from adult patients in Russia in 2024–2025.

## 2. Materials and Methods

A non-interventional, observational, cross-sectional study was conducted at the Smorodintsev Research Institute of Influenza of the Ministry of Health, St. Petersburg, Russia, from January 2024 to September 2025.

### 2.1. Clinical Samples

A total of 1888 clinical samples obtained from adult HIV-infected patients from 6 federal districts of the Russian Federation were included in this study (Table 1). These samples were selected from 2250 collected HIV-positive specimens according to a viral load greater than 3 log10 copies/mL.

The average median age was 42.08 years (IQR 36.69–47.95 years). The proportion of males was 60.86% (*n* = 1149), and that of females was 39.14% (*n* = 739). In most cases HIV infection was transmitted heterosexually (52.13%), less often through intravenous drug use (27.32%). Advanced stages of HIV infection (4A–4C) according to Pokrovsky’s classification [4] were observed in more than half of the patients. In 41.51% of cases (*n* = 494), stage 3 HIV infection was indicated in the clinical diagnosis. In all examined samples (blood plasma) HIV-1 RNA was detected by RT-PCR. A viral load greater than 4 log10 copies/mL was observed in 90.37% of cases.

HIV-infected patients (77.65%, *n* = 1466) having information about their ART experience were selected for this study and were divided into two groups: 28.04% (*n* = 411) had never received ART, and 71.96% (*n* = 1055) had received ART. The vast majority of patients with ART experience (88.34%, *n* = 932) were receiving ART at the time of collection of the biological sample. The most frequently prescribed ART regimens for patients included: NRTIs—lamivudine (3TC) (94.96%, *n* = 885) and tenofovir (TDF) (83.37%, *n* = 777); NNRTI—efavirenz (EFV) (37.02%, *n* = 345); INSTI—dolutegravir (DTG) (16.74%, *n* = 156); and PIs—lopinavir (LPV) (8.58%, *n* = 80) and darunavir (DRV) (6.33%, *n* = 59).

### 2.2. Extraction and Viral Load Quantification

RNA extraction was performed from 200 µL of plasma using the Amplisens “Magno-Sorb” (Amplisens, Moscow, Russia) and Ampliprime “MagnoPrime Ultra” (Nextbio, Moscow, Russia) kits. The viral load in samples was determined using the AmpliPrime HIV kit (Nextbio, Moscow, Russia) for quantitative real-time RT-PCR according to the manufacturer’s protocol.

### 2.3. Obtaining Consensus Sequences of the HIV-1 pol and env Genes

Amplification of the HIV-1 *pol* and *env* genes was performed using nested PCR. Each gene was covered by a single separate amplicon. For the first stage the BioMaster RT-PCR–Premium (2×) kit (Biolabmix, Novosibirsk, Russia) was used for reverse transcription and subsequent PCR in the same reaction with two pairs of HIV-specific outer primers according to the manufacturer’s instructions (Pol-F-1 and Pol-R-1 for *pol* gene and Env-F-1 and Env-R-1 for *env* gene). The second round of nested PCR was performed with two pairs of inner HIV-specific primers (Pol-F-2 and Pol-R-2 for *pol* gene and Env-F-2 and Env-R-2 for *env* gene) using the BioMaster LR HS-PCR (2×) kit (Biolabmix, Novosibirsk, Russia).

**Figure 1:**
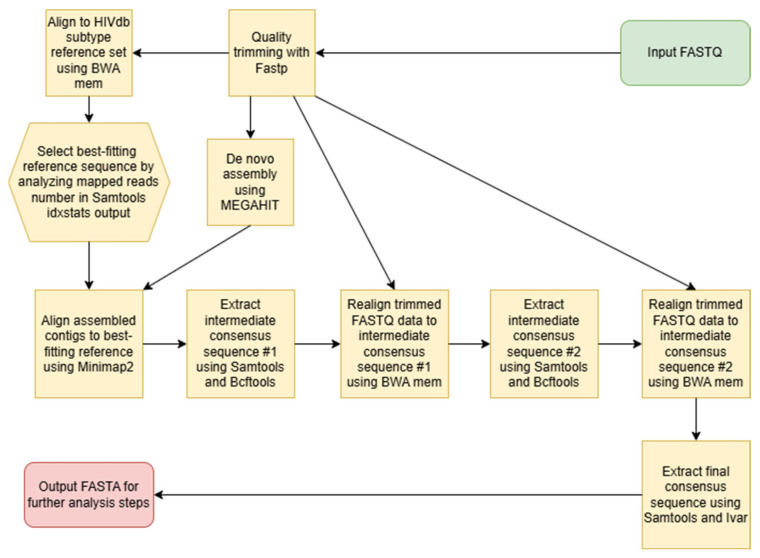
NGS data analysis pipeline structure.

The resulting PCR products corresponded to genomic fragments with coordinates 2252-5075 and 6207-7952 (HIV-1 strain HXB2, GenBank #K03455). The positions of the amplicons in the HIV-1 reference genome are shown in Figure 1. Primer sequences are shown in Table 2.

Sequencing of the HIV-1 *pol* and *env* gene amplification products was performed by next-generation sequencing using DNBSEQ-G400 (MGI, Shenzhen, China) and Illumina NextSeq 2000 (Illumina, San Diego, CA, USA) instruments according to the manufacturer’s instructions. Libraries were prepared for the MGI sequencing platform using the Fast PCR-FREE FS DNA Library Prep Set (MGI, Shenzhen, China) and for the Illumina sequencing platform using the Illumina DNA Prep kit (Illumina, San Diego, CA, USA).

The resulting data contained at least 100,000 reads per sample. Data assembly was performed using a custom Python script (version 3.12) resembling the methodology of the Shiver tool [27]. The data analysis pipeline structure is shown in Figure 1. At first these reads were quality trimmed using FastP software (version 1.0.1) [28]. Then trimmed data was used for de novo assembly using MEGAHIT assembler [29]. In parallel, trimmed data was mapped to a subtype reference set of sequences obtained from the Los Alamos HIV Sequence database using the BWA mem algorithm [30]. The best-fitting reference was determined using the output of the Samtools idxstats [31] command as having the largest number of mapped reads. Then contigs produced by MEGAHIT were aligned to the best-fitting reference using Minimap2 [32], and the majority rule consensus was extracted from the alignment using a combination of Samtools and BCFtools [31]. This intermediate consensus #1 was used for the second mapping step using the BWA mem algorithm. The consensus sequence from this alignment was extracted using a combination of Samtools and BCFtools (including positions with coverage depth greater than 10, single-nucleotide polymorphisms (SNPs) with allelic frequency greater than 50%, and indels with allelic frequency greater than 75%) and used as consensus sequence #2. The intermediate consensus sequence #2 was used for the third mapping step using the BWA mem algorithm, and final consensus sequences were generated using a combination of Samtools and the Ivar consensus command with default parameters [33]. The analysis was performed separately for *pol* and *env* genes.

### 2.4. HIV-1 Subtyping Using pol Gene Sequences

Assembled consensus sequences of the *pol* gene were used for HIV-1 subtyping. Determination of HIV-1 subtypes was performed using specific tools: the COMET web tool [34], a local Docker-based installation of the Stanford University Sierra tool (version 9.7) [35], and the Nextclade web tool [36]. If at least two of three tools gave the same subtype prediction, the sequence was considered subtyped. If all three tools gave different predictions, the sequence was considered unclassified. For CRF63_02A6 in cases when COMET or Nextclade predicted CRF63_02A6 and Sierra predicted A6 + CRF02_AG the final subtype was considered as CRF63_02A6. For CRF03_A6B in cases when COMET or Nextclade predicted CRF03_A6B and Sierra predicted A6 + B the final subtype was considered as CRF03_A6B.

### 2.5. Analysis of Drug Resistance Mutations in HIV-1 pol Gene

The nucleotide sequences of the *pol* gene encoding HIV-1 protease, reverse transcriptase, and integrase were analyzed using a local Docker-based installation of the Stanford University HIVdb database (version 9.7) [37]. The database was used for determining of resistance mutations including predicted levels of DR to antiretroviral drugs in accordance with the WHO recommendations: the list of surveillance drug resistance mutations (SDRMs) of 2009 [38], and the list of SDRMs to HIV-1 integrase inhibitors of 2019 [39]. Predicted HIV-1 resistance rates were determined for 26 antiretroviral drugs including PIs: atazanavir (ATV), lopinavir (LPV), darunavir (DRV), Fosamprenavir (FPV), Nelfinavir (NFV), Saquinavir (SQV), Tipranavir (TPV), Indinavir (IDV); NRTIs: lamivudine (3TC), Abacavir (ABC), Stavudine (D4T), Didanosine (DDI), emtricitabine (FTC), tenofovir (TDF), zidovudine (AZT); NNRTIs: Doravirine (DOR), efavirenz (EFV), Etravirine (ETR), Rilpivirine (RPV), Nevirapine (NVP), Dapivirine (DPV); and INSTIs: Bictegravir (BIC), dolutegravir (DTG), Cabotegravir (CAB), Elvitegravir (EVG), Raltegravir (RAL). Potentially low-level drug resistance was considered as the absence of HIV-1 resistance to the drug.

All sequences included in this study met the validation criteria for three regions encoding protease, reverse transcriptase, and integrase. Nucleotide sequences containing uncovered positions corresponding to the known DRMs in HIV-1 protease, reverse transcriptase, and integrase were excluded from the analysis. Furthermore, sequences containing more than 2 stop codons + insertions/deletions + highly ambiguous nucleotides in the protease-encoding sequence, more than 4 in the reverse transcriptase sequence, and more than 3 in the integrase sequence were not included. The maximum allowed number of apolipoprotein B mRNA editing catalytic polypeptide (APOBEC) 3G/F amino acid substitutions in protease, reverse transcriptase, and integrase was 2, 3, and 3 substitutions, and the maximum allowed number of highly uncommon mutations was 8, 15, and 10 mutations, respectively.

### 2.6. Analysis of Consensus Sequences of the HIV-1 env Gene and CXCR4 Cell Tropism Prediction

The obtained consensus nucleotide sequences of the *env* gene encoding HIV-1 envelope protein gp120 were used for analysis of the amino acid sequence of the HIV-1 gp120 V3-loop, which is a major determinant of HIV’s cell tropism, dictating whether the virus primarily infects T cells (binding to CXCR4 co-receptor) or macrophages (binding to CCR5 co-receptor). Previous benchmarks [40] demonstrated that machine learning-based methods of HIV-1 cell tropism prediction for A and C subtypes do not leave behind similar empiric rules. So, a custom Python script implementing a set of empiric rules was developed [41,42,43]. Briefly, the amino acid sequence of the gp120 V3-loop was extracted from the output file of Sierra and checked according to classification rules in Raymond et al. [42,44]. The results obtained with empirical rules were compared with the predictions of the Geno2pheno [coreceptor] tool (version 2.5) using amino acid sequences of the gp120 V3-loop extracted from the output of Sierra [44]. Partial sequences and sequences containing ambiguous positions (X) were excluded from both analyses. The false positive rate (FPR) threshold for Geno2pheno [coreceptor] was set to 10% according to recommendations from the European Consensus Group on clinical management of HIV-1 tropism testing.

### 2.7. Statistical Analysis

The collected data was cleaned, formalized, and analyzed using the R software environment (version 4.5.1, 13 June 2025). The median (Me) was used as a measure of central tendency to describe quantitative indicators, and the lower (LQ) and upper (HQ) quartiles were calculated as measures of data variability. Group nominal indicators are presented as feature frequencies in absolute (Abs., units) and relative (%) values. To assess differences between samples for nominal features, contingency tables were created and the Pearson chi-square test or two-tailed Fisher’s exact test was performed. The Holm method was used to adjust for multiple hypothesis testing. The significance level was set at α ≤ 0.05.

## 3. Results

### 3.1. Testing of the NGS Protocol for HIV-1 pol and env Genes

**Figure 2:**
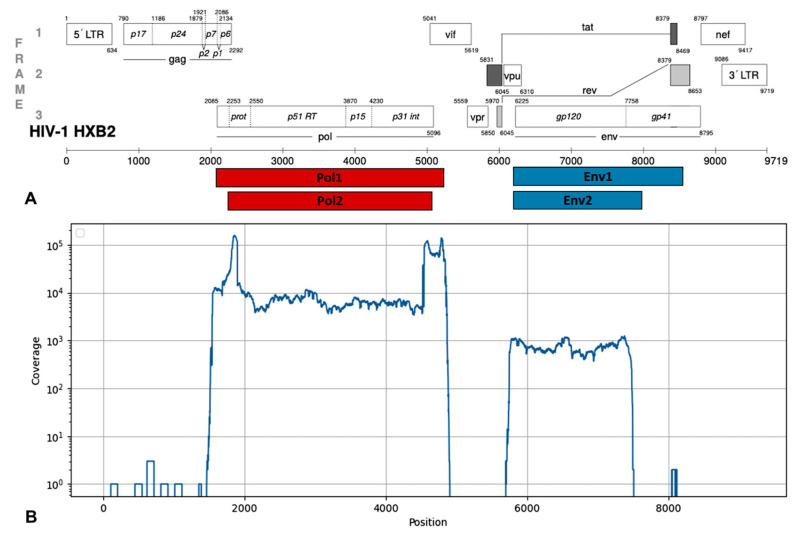
The amplicon layout on the HIV-1 genome (**A**) (modified from [45]) and a typical coverage depth diagram (**B**).

In total, 1888 samples with a viral load in plasma starting from 3 log10 copies/mL were studied. Of these, 1521 samples were tested with primers for both the *pol* and *env* genes and 367 samples were tested only with primers for the *pol* gene. In all, 1888 (100%) sequences suitable for further analysis were obtained for the *pol* gene and 916 (60%) sequences were obtained for the *env* gene. PCR products for the *pol* gene had coordinates 2252-5075 in HXB2 reference HIV-1 genome (GenBank accession number NC_001802). PCR products for the *env* gene had coordinates 6207-7952 in HXB2 reference HIV-1 genome (GenBank accession number NC_001802) (Figure 2A). The median coverage of the *pol* gene was about 5000, and that of the *env* gene was 700. A typical coverage distribution for a sample with both gene sequences is shown in Figure 2B.

Assembled nucleotide sequences of *pol* genes were deposited into the Russian HIV Drug Resistance database RuHIV (https://ruhiv.ru/, accessed on 26 January 2026). The accession numbers of deposited sequences are listed in the Data Availability Statement.

### 3.2. Subtyping of HIV-1 Viruses

**Figure 3:**
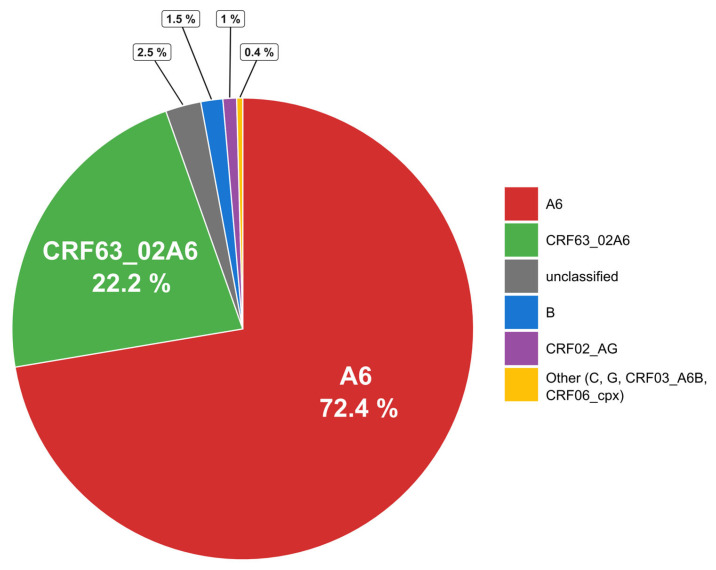
Distribution of HIV-1 subtypes in analyzed samples.

Subtyping of HIV-1 showed the prevalence of the A6 sub-subtype (72.4% of all subtyped samples). Circulating recombinant form CRF63_02A6 was the second most prevalent at 22.2%. Subtype B (1.5%) and recombinant CRF02_AG (1%) were also found. Subtypes C and G, recombinants CRF03_A6B and CRF06_cpx, represented about 0.4% of the studied samples in total. Of the samples 2.5% were considered unclassified as they could not be subtyped by the set of tools used (see Section 2.4). The subtype distribution is shown in Figure 3.

**Figure 4:**
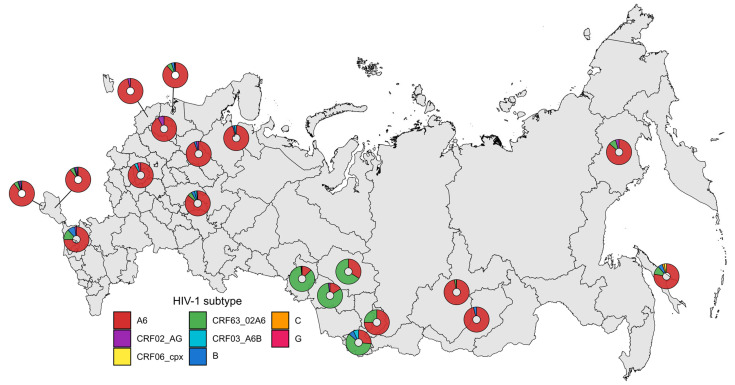
Geographical distribution of HIV-1 subtypes across Russia.

The geographic distribution of HIV-1 subtypes across Russia was heterogeneous. Sub-subtype A6 dominated in the European, Eastern Siberian and Far Eastern regions. Recombinant CRF63_02A6 dominated in the Western Siberian region. The distribution of HIV-1 subtypes of analyzed samples is shown in Figure 4.

### 3.3. Analysis of Drug Resistance Mutations

In the HIV-1 protease sequence, SDRMs were observed extremely rarely, among which the most common were M46I, K43T, and L33F (Table 3). The surveillance mutation M46I is known to increase the catalytic activity of the protease and is associated with reduced susceptibility to ATV and LPV. In turn, the K43T and L33F substitutions are non-polymorphic accessory mutations also associated with DR to ATV, LPV, and DRV. In isolated cases, SDRMs such as I54S/M, F53L, I47V, I54V, and M46L were observed in HIV-1 protease, primarily in patients with ART experience. The F53L mutation is a non-polymorphic accessory mutation that, in combination with others, leads to reduced susceptibility of HIV-1 to ATV. Non-polymorphic mutations I54M and I54S are associated with a decrease in the susceptibility of the virus to LPV, ATV and DRV, and I54S is found predominantly in viruses with multidrug resistance to PI.

In the HIV-1 reverse transcriptase sequence, the most common NRTI DRMs were M184V/I and K65R (Table 4). These SDRMs, individually and in combination, are the most common NRTI mutations arising in patients treated with first-line ART. The M184V and M184I mutations reduce HIV-1 susceptibility to 3TC/FTC by more than 200-fold, reduce susceptibility to ABC by 3-fold, and, conversely, increase viral susceptibility to AZT and TDF. In turn, the K65R mutation is accompanied by a decrease in HIV-1 susceptibility to 3TC, FTC, TDF, and ABC. The frequently occurring non-polymorphic amino acid substitution A62V and the polymorphic S68G are likely mutations of adaptation and correct the viral replication deficiency associated with the K65R substitution and the Q151M and T69 insertions. The most common NNRTI DRMs were K103N, V90I, E138A, G190S, K101E, and Y181C (Table 4). The SDRM K103N is one of the most frequently transmitted non-polymorphic mutations conferring resistance to ART drugs. This amino acid substitution significantly reduces HIV-1 susceptibility to NVP and EFV but does not reduce the susceptibility to RPV, ETR, or DOR. The E138A polymorphic mutation is associated with reduced HIV-1 susceptibility to ETR, while the G190S and Y188L non-polymorphic mutations are associated with a marked reduction in susceptibility to NVP and EFV. The non-polymorphic SDRM K101E is frequently found in combination with other NNRTI resistance mutations and reduces susceptibility to NVP by 3–10 times and to EFV, ETR, and RPV by approximately 2-fold. The additional polymorphic mutation V90I does not significantly reduce viral susceptibility to any NNRTI drug but is frequently detected in patients receiving RPV and less frequently in patients receiving NVP and EFV. A comparative analysis of the frequencies of DRMs in HIV-1 reverse transcriptase revealed that the vast majority of observed amino acid substitutions were associated with ART. Meanwhile, the S68G and E138A mutations were equally common among both treatment-naïve and ART-treated patients. SDRMs to NRTIs—K65R and M184V/I—and NNRTIs—G190S, K101E and Y181C—were practically not found among HIV-infected patients without ART experience.

In the HIV-1 integrase sequence, the most frequently observed DRMs were L74I, E157Q, T97A, and Y143R (Table 5). For HIV-1 subtype A, isoleucine at position 74 of the integrase is a consensus amino acid. The L74I substitution was observed in the majority of the studied samples. Among treatment-experienced patients this substitution was observed significantly more frequently (*p* = 0.013) and was defined as the major mutation for subtype A6 associated with the usage of CAB (INSTI) [46]. The proportions of E157Q, T97A, and Y143R mutations did not exceed 1–2% and did not depend on ART experience in patients. The E157Q amino acid substitution does not independently affect viral susceptibility to ART drugs and is often found in combination with other resistance mutations. In turn, the T97A polymorphic mutation reduces susceptibility to EVG by approximately threefold and, when combined with other INSTI resistance mutations, can significantly reduce the virus’s susceptibility to each of them. In isolated cases, surveillance mutations conferring DR to DTG, such as R263K (*n* = 3), T66K (*n* = 1), G118R (*n* = 2), and Q148R (*n* = 4), were observed in the amino acid sequence of HIV-1 integrase.

The most common combinations of SDRMs were combinations of two or three amino acid substitutions in the reverse transcriptase sequence. Sequences obtained from patients without ART experience very rarely contained more than one SDRM. In isolated cases, combinations such as G190S + K101E (0.73%, *n* = 3), G190S + M184V (0.73%, *n* = 3), K101E + M184V (0.73%, *n* = 3) and G190S + K101E + M184V (0.73%, *n* = 3) were observed. In contrast, among HIV-infected patients with ART experience, combinations of two or more SDRMs in the reverse transcriptase were observed significantly more frequently: G190S + K101E (5.78%, *n* = 61), G190S + M184V (5.40%, *n* = 57), K65R + M184V (5.40%, *n* = 57), K103N + M184V (5.21%, *n* = 55), G190S + Y181C (5.21%, *n* = 55) and G190S + K65R (5.40%, *n* = 57). The most common combinations of three surveillance mutations in the group of patients with ART experience were G190S + K65R + Y181C (3.51%, *n* = 37), G190S + K101E + Y181C (3.51%, *n* = 37), G190S + K101E + M184V (3.32%, *n* = 35) and G190S + K101E + K65R (3.13%, *n* = 33).

The list of antivirals affected by the most common key DRMs (occurrence higher than 0.5% for protease and integrase inhibitors and higher than 5% for reverse transcriptase inhibitors) is shown in Table 6 (according to [46]). Cases when a substitution occurs at the important position but leads to an amino acid other than those in recognized DRMs are shown in italic.

### 3.4. Cell Co-Receptor Tropism Prediction

After NGS sequence assembly and V3-loop amino acid sequence translation with the Sierra tool, 916 amino acid sequences were obtained. Other samples were excluded from the analysis due to lower coverage of the V3 region and the presence of ambiguous positions after the translation. The analysis of calculated amino acid sequences of the HIV-1 gp120 V3-loop showed that 866 viruses had no signs of possible CXCR4 tropism. In all, 50 (5.4%) samples were classified as having possible CXCR4 tropism by at least one empirical rule. Different rules predicted possible CXCR4 co-receptor tropism in different samples. The results of prediction are shown in Table 7.

Spontaneous emergence of CXCR4 co-receptor tropism along the course of HIV infection is described in the literature [47,48,49]. More than a half of the analyzed samples were collected from patients with late stages of HIV infection, but the proportion of possible CXCR4 co-receptor tropism was significantly lower. At the moment, no significant correlations among empirical rule, disease stage and used ART have been found.

After empirical rule co-receptor tropism prediction, all samples were divided into six groups according to the prediction results. Each group was analyzed using the Geno2pheno [coreceptor] web tool separately. The Geno2pheno [coreceptor] algorithm predicted 175 (19% of all sequences) samples with possible CXCR4 co-receptor tropism in total. The concordance between the empirical rule and Geno2pheno [coreceptor] predictions is shown in Table 8.

Higher concordance between predictions by Geno2pheno [co-receptor] and empirical rules was observed for charge- and glycosylation-based rules. The widely used 11/25 rule showed lower concordance.

## 4. Discussion

Monitoring the emergence and patterns of antiretroviral drug resistance is crucial for the success and sustainability of treatment programs. The developed NGS-based protocol allows for performing the analysis of a large number of samples in a shorter period of time and at a lower cost per sample compared to Sanger sequencing-based protocols, contributing to strengthening the capacity of surveillance of HIV-1 ART resistance and cell co-receptor tropism [20,22,24]. NGS could improve HIV genotyping to guide treatment decisions for enhancing ART efficacy [13].

A large-scale study was conducted to assess the prevalence of HIV-1 DR and multidrug resistance mutations to four classes of antiretroviral drugs simultaneously in two groups of patients, depending on their ART experience. Taking into account the constant dynamics of HIV-1 DR, including that associated with the emergence of new viral variants in circulation and changes in first-line ART regimens, our data allows us to update and expand the existing knowledge in this area. Distinguishing by ART status, the prevalence of HIV-1 DR was found to be lower for patients without ART experience and higher for ART-experienced individuals, as in other studies [13,50,51,52]. The results of our study are consistent with the results of other authors [7,50,51,53] and indicate a high prevalence of HIV-1 drug resistance to NNRTI and NRTI drugs among patients with ART experience. Interestingly, NNRTIs remain the drug class with the highest prevalence of transmitted DRMs [13]. The most frequently encountered SDRMs were amino acid substitutions in the reverse transcriptase sequence, M184V and K103N, with the latter being quite common among patients without ART experience. High levels of HIV-1 resistance to the most commonly used NNRTI (EFV) and NRTI (3TC, ABC) drugs in ART regimens were frequently observed. Similar occurrence of ART associated with HIV DR was observed in other countries. The distribution of resistance to NRTIs among ART-experienced patients from Portugal, Portuguese-speaking African countries and Brazil according to the type of ART indicated TDF showing the lowest resistance, compared to FTC, 3TC, and ABC with higher resistance. NNRTI resistance in ART-experienced patients with EFV, NVP and DOR was at a medium level [51]. Nevertheless, treatment-experienced individuals showed significantly higher resistance rates, especially to NRTIs, NNRTIs, and INSTIs, in Italy [52] and Uganda [21]. Pre-treatment DR prevalence for NNRTIs and low levels for NRTIs and INSTIs were observed in Southeastern China [54]. Among patients without ART experience, the highest levels of DR were observed for NNRTI drugs (EFV, NVP, RPV), which indicates possible consolidation of the corresponding resistance mutations in the reverse transcriptase sequence in the viral population, including additional adaptation mutations such as A62V, S68G and E138A. Despite the fact of low detection levels of polymorphic accessory INSTI resistance mutations E157Q and T97A that do not depend on ART experience, an increase in the number of these SDRMs over time was found worldwide [39]. However, a minimal level of resistance mutations to INSTIs in patients without ART experience and a high level of resistance mutations in ART-exposed patients were observed in Portugal [51]. Analysis of the sequences of three HIV-1 *pol* gene fragments encoding protease, reverse transcriptase, and integrase simultaneously allowed us to estimate the prevalence of HIV-1 multidrug resistance to different classes of antiretroviral drugs, including combinations of individual SDRMs. Combinations of two or three amino acid substitutions were identified as the most common combinations of SDRMs among patients with ART experience (G190S + K101E, G190S + M184V, K65R + M184V, K103N + M184V, G190S + K65R + Y181C, G190S + K101E + Y181C, and G190S + K101E + M184V). It is noteworthy that these combinations contain substitutions associated with the development of drug resistance to two classes of antiretroviral drugs simultaneously: NRTIs and NNRTIs. Similar combinations of at least K103N and G190A, SDRMs that increased the drug resistance scores to the existing NNRTIs, were identified by other authors [21,51]. Patients without ART experience, especially those previously INSTI-naïve while receiving DTG, developed INSTI-associated SDRMs, including R263K, G118R, N155H, and Q148H and their combinations [55]. The most common combination of HIV-1 multidrug resistance to different classes of ART was NRTI + NNRTI resistance. Combinations of HIV-1 drug resistance to NRTIs + INSTIs, NNRTIs + INSTIs, and NRTIs + NNRTIs + INSTIs were observed significantly less frequently among ART-experienced patients. Highly ART-experienced patients, especially in suboptimal combinations of ART classes, can develop resistance-associated mutation patterns, conferring multidrug cross-resistance to all widely available ARTs and their combinations [12,56,57]. The most frequently observed surveillance mutations conferring drug resistance were amino acid substitutions in the reverse transcriptase sequence—M184V and K103N—with the latter being quite common among ART-inexperienced patients. On the contrary, the M184V mutation in the reverse transcriptase was found as one of those resulting in increased resistance to NRTIs (3TC) but with low-level resistance to other ART drugs [58], and the K103N SDRM was considered the most prevalent mutation conferring higher resistance to NNRTIs in treatment-experienced patients [51,52]. High levels of HIV-1 resistance were frequently observed for the most commonly used NNRTIs (EFV) and NRTIs (3TC, ABC) in ART regimens, which may be explained by targeted viral selection under drug pressure in the patient. Among ART-inexperienced patients the highest levels of drug resistance were observed for NNRTIs (EFV, NVP, RPV), suggesting the possible persistence of the corresponding resistance mutations in the reverse transcriptase sequence, including additional mutations of adaptation such as A62V, S68G, and E138A. Two mutations, A62V (NRTI-associated) and E138A (NNRTI-associated), were shown to be the most common in treatment-naïve Russian patients, while S68G substitution was associated with TDF (NRTI) use. All these mutations are predominantly found in subtype A6 HIV, which is the prevailing genetic variant in Russia [50,59]. The results of our study are consistent, showing the prevalence of subtype A6 (72.4%). Despite the higher proportion of samples collected from patients with advanced stages of HIV-1 infection, viruses with signs of possible CXCR4 tropism were observed more rarely. In our study CXCR4 tropism was predicted using five separate empirical rules, resulting in five independent sets of possible CXCR4-tropic viruses. The Geno2pheno [coreceptor] web tool predicted possible CXCR4 co-receptor tropism in a larger number of samples. But different previous studies showed various levels of reliability and correctness of genotypic methods predicting CXCR4 tropism, depending on the HIV-1 subtype [40,60]. Due to the lack of phenotypic data for non-B subtypes (especially subtype A6 actively circulating in former USSR countries) the majority of widely used predictive algorithms (like Geno2pheno [coreceptor]) were developed mostly based on data obtained for subtype B HIV-1, while in our study this subtype was observed only in 1.5% of cases. So, there remains a need to further investigate and develop better predictive algorithms, especially for multiple non-B HIV-1 subtypes circulating outside of Western Europe and the USA [13,61].

## 5. Conclusions

According to the results of our study the newly developed NGS-based protocol can be used for the detection and monitoring of HIV-1 subtype diversity, ART resistance including multidrug resistance mutations, and cell co-receptor tropism. The usage of next-generation sequencing platforms instead of the previously used Sanger sequencing can make the analysis cheaper and faster. Further development of data processing pipelines can enhance the analysis by investigating viral population dynamics and producing more valuable predictions from the same data generated by already-established laboratory protocols.

## Figures and Tables

**Table 1:** Distribution of HIV-infected patients by federal district.

Federal District	Total Samples (*n* = 1888) Abs./%
Siberian	732/38.77
Southern	551/29.18
Volga region	258/13.67
Northwestern	175/9.27
Far Eastern	155/8.21
Central	17/0.90

**Table 2:** Primer sequences used in this study.

Primer Name	Primer Sequence
Pol-F-1	GGGCCCCTAGGAAAAAGGG
Pol-R-1	CCTGTATGCAGACCCCAATATGTT
Pol-F-2	CCCTCARATCACTCTTTGGCA
Pol-R-2	TGCCACACAATCATCACCTG
Env-F-1	GAGCAGAAGAYAGTGGMAATGA
Env-R-1	GMKGAARAGGCACAGGYTCC
Env-F-2	GAGCAGAAGAYAGTGGMAATGA
Env-R-2	GAGCTGYTTRATGCCCCAGAC

**Table 3:** The most common drug resistance mutations in the amino acid sequence of HIV-1 protease.

Mutation	Total (*n* = 1888) Abs./%	ART Experience (*n* = 1466)		
		No (*n* = 411) Abs./%	Yes (*n* = 1055) Abs./%	*p*
M46I ^1^	15/0.79	4/0.97	10/0.95	1.000
K43T	10/0.53	2/0.49	5/0.47	1.000
L33F	10/0.53	1/0.24	4/0.38	1.000
Q58E	6/0.32	0/0.00	5/0.47	1.000
V11I	4/0.21	0/0.00	2/0.19	1.000
I54S ^1^	4/0.21	1/0.24	2/0.19	1.000
F53L ^1^	3/0.16	0/0.00	2/0.19	1.000
I47V ^1^	2/0.11	0/0.00	2/0.19	1.000
I54V ^1^	2/0.11	0/0.00	1/0.09	1.000
M46L ^1^	2/0.11	1/0.24	1/0.09	1.000

^1^ SDRM.

**Table 4:** The most common drug resistance mutations in the amino acid sequence of HIV-1 reverse transcriptase.

Mutation	Total (*n* = 1888) Abs./%	ART Experience (*n* = 1466)		
		No (*n* = 411) Abs./%	Yes (*n* = 1055) Abs./%	*p*
A62V	506/26.80	64/15.57	334/31.66	<0.001
M184V ^1^	234/12.39	6/1.46	210/19.91	<0.001
K103N ^1^	206/10.91	28/6.81	149/14.12	0.013
S68G	176/9.32	32/7.79	114/10.81	1.000
V90I	162/8.58	20/4.87	115/10.90	0.037
E138A	158/8.37	31/7.54	91/8.63	1.000
G190S ^1^	135/7.15	7/1.70	116/11.00	<0.001
K65R ^1^	114/6.04	0/0.00	105/9.95	<0.001
V106I	113/5.99	9/2.19	93/8.82	0.001
K101E ^1^	108/5.72	5/1.22	95/9.00	<0.001
Y181C ^1^	87/4.61	2/0.49	76/7.20	<0.001
M184I ^1^	58/3.07	1/0.24	54/5.12	<0.001
H221Y	46/2.44	1/0.24	41/3.89	0.002
V179E	42/2.22	4/0.97	29/2.75	1.000
E138K	36/1.91	0/0.00	31/2.94	0.004
P225H ^1^	34/1.80	1/0.24	31/2.94	0.037
Y115F ^1^	31/1.64	0/0.00	28/2.65	0.012
D67N ^1^	29/1.54	2/0.49	26/2.46	0.675
Y318F	28/1.48	0/0.00	26/2.46	0.019

^1^ SDRMs.

**Table 5:** The most common drug resistance mutations in the amino acid sequence of HIV-1 integrase.

Mutation	Total (*n* = 1888) Abs./%	ART Experience (*n* = 1466)		
		No (*n* = 411) Abs./%	Yes (*n* = 1055) Abs./%	*p*
L74I	1422/75.32	275/66.91	802/76.02	0.013
E157Q	30/1.59	3/0.73	21/1.99	1.000
T97A	12/0.64	0/0.00	9/0.85	1.000
Y143R ^1^	7/0.37	0/0.00	7/0.66	1.000
G163R	4/0.21	1/0.24	3/0.28	1.000
E138K ^1^	4/0.21	0/0.00	4/0.38	1.000
Q148R ^1^	4/0.21	0/0.00	3/0.28	1.000
D232N	4/0.21	0/0.00	3/0.28	1.000
L74M	4/0.21	0/0.00	2/0.19	1.000
N155H ^1^	3/0.16	0/0.00	3/0.28	1.000
R263K ^1^	3/0.16	0/0.00	2/0.19	1.000
E92G ^1^	3/0.16	2/0.49	1/0.09	1.000
G140A ^1^	2/0.11	0/0.00	1/0.09	1.000
Y143H ^1^	2/0.11	0/0.00	2/0.19	1.000
G118R ^1^	2/0.11	0/0.00	2/0.19	1.000
T66I ^1^	2/0.11	1/0.24	1/0.09	1.000
E92Q ^1^	2/0.11	0/0.00	2/0.19	1.000
S147G ^1^	2/0.11	0/0.00	2/0.19	1.000

^1^ SDRMs.

**Table 6:** Antiviral drugs affected by drug resistance mutations found in Russian patients.

Protein	Drug Class	Drug Name	Resistance Mutations
Protease	PIs	Atazanavir	L33F, M46I
		Tipranavir	L33F, K43T, M46I
Reverse transcriptase	NRTIs	Abacavir	K65R, M184V
		Emtricitabine/Lamivudine	K65R, M184V
		Tenofovir	K65R
	NNRTIs	Doravirine	V106I, *G190S*
		Efavirenz	*K101E*, K103N, *V106I*, G190S
		Etravirine	V90I, K101E, E138A, G190S
		Nevirapine	*K101E*, K103N, *V106I*, *G190S*
		Rilpivirine	K101E, E138A
Integrase	INSTIs	Cabotegravir	L74I, T97A

**Table 7:** Positive CXCR4 co-receptor tropism prediction results using empirical rules.

Rule	Number of Positive Samples (Proportion from the Total Number of Samples)	Disease Stages ^1^ of Studied Patients	Used Antiretroviral Therapy
A net charge rule of ≥+5 and total number of charged amino acids in the V3-loop of ≥8	23 (2.5%)	3, 4A, 4B, 4C	3TC:ABC:LPV:RTV; 3TC:AZT:ATV:RTV; 3TC:LPV:RTV:TDF; 3TC:DTG:TDF; 3TC:EFV:TDF; FTC:RPV:TDF
Loss of the N-linked glycosylation site in the V3-loop and a net charge of ≥+4	11 (1.2%)	3, 4A	3TC:ATV:RTV:TDF; 3TC:AZT:LPV:RTV; 3TC:EFV:TDF
R or K at position 11 and/or K at position 25 of the V3-loop (11/25 rule)	10 (1.1%)	3, 4A, 4C	3TC:EFV:TDF 3TC:LPV:RTV:TDF
R at position 25 of the V3-loop and a net charge of ≥+5	5 (0.5%)	4A, 4B	3TC:ABC:DTG; 3TC:ABC:ESV; 3TC:ABC:LPV:RTV; 3TC:DTG:TDF
Net charge ≥+6	1 (0.1%)	2B	no data

^1^ According to Pokrovsky’s classification [4].

**Table 8:** Comparison between empirical rule and Geno2pheno [coreceptor] CXCR4 co-receptor tropism predictions.

Rule	Number of Samples in Group	Number of Geno2Pheno Results Matching Empirical Predictions	Percentage of Concordance Between Geno2Pheno and Empirical Predictions
No CXCR4 co-receptor tropism predicted	866	728	84.0%
A net charge rule of ≥+5 and total number of charged amino acids in the V3-loop of ≥8	23	21	91.3%
Loss of the N-linked glycosylation site in the V3-loop and a net charge of ≥+4	11	9	81.8%
R or K at position 11 and/or K at position 25 of the V3-loop (11/25 rule)	10	4	40%
R at position 25 of the V3-loop and a net charge of ≥+5	5	3	60%
Net charge ≥+6	1	0	0%

## Data Availability

All nucleotide sequence data generated in this study is available in the Russian HIV Drug Resistance database RuHIV (https://ruhiv.ru/, accessed on 26 January 2026). Accession numbers: RHD36518-RHD36522, RHD36535-RHD36557, RHD36559-RHD36562, RHD36564-RHD36565, RHD36567-RHD36571, RHD36573-RHD36581, RHD36583-RHD36637, RHD36639-RHD36641, RHD36644-RHD36645, RHD36647-RHD36648, RHD36650-RHD36653, RHD36655, RHD36658-RHD36659, RHD36664, RHD36672, RHD36674-RHD36683, RHD36685-RHD36686, RHD36688-RHD36720, RHD36722-RHD36736, RHD36739-RHD36745, RHD36747-RHD36752, RHD36754, RHD36756-RHD36795, RHD36797-RHD36808, RHD36810, RHD36812, RHD36814-RHD36829, RHD36831-RHD36889, RHD36891-RHD36896, RHD36899-RHD36915, RHD36917-RHD36923, RHD36925, RHD36927-RHD36935, RHD36937-RHD36969, RHD36971-RHD36990, RHD36992-RHD36994, RHD36996-RHD36999, RHD37001-RHD37004, RHD37006-RHD37007, RHD37009-RHD37018, RHD37020, RHD37024-RHD37025, RHD37027-RHD37029, RHD37031, RHD37033-RHD37034, RHD37036-RHD37038, RHD37040, RHD37043-RHD37053, RHD37055-RHD37063, RHD37065-RHD37070, RHD37072-RHD37074, RHD37076, RHD37078-RHD37084, RHD37086, RHD37090, RHD37092-RHD37104, RHD37106-RHD37107, RHD37109-RHD37111, RHD37113, RHD37116-RHD37117, RHD37119-RHD37133, RHD37135-RHD37148, RHD37150-RHD37153, RHD37155-RHD37163, RHD37165-RHD37167, RHD37169-RHD37174, RHD37178-RHD37182, RHD37184, RHD37187-RHD37197, RHD37199-RHD37218, RHD37221-RHD37223, RHD37227-RHD37233, RHD37235-RHD37255, RHD37257-RHD37269, RHD37271-RHD37283, RHD37285-RHD37289, RHD37293, RHD37295-RHD37391, RHD37393-RHD37397, RHD37399-RHD37430, RHD37432-RHD37529, RHD37531-RHD37537, RHD37539, RHD37541-RHD37546, RHD37548-RHD37577, RHD37579-RHD37614, RHD37616-RHD37638, RHD37640-RHD37703, RHD37705-RHD37710, RHD37712-RHD37761, RHD37763-RHD37775, RHD37777-RHD37785, RHD37787-RHD37793, RHD37796-RHD37808, RHD37810-RHD37836, RHD37838-RHD37849, RHD37851-RHD37853, RHD37855-RHD37860, RHD37862-RHD37864, RHD37866-RHD37887, RHD37889, RHD37891-RHD37905, RHD37907-RHD37919, RHD37921-RHD37944, RHD37946-RHD37953, RHD37955-RHD37976, RHD37978-RHD37984, RHD37986-RHD37989, RHD37991-RHD37993, RHD37995-RHD38010, RHD38013-RHD38017, RHD38019-RHD38036, RHD38038-RHD38045, RHD38047-RHD38050, RHD38052-RHD38073, RHD38075-RHD38076, RHD38078-RHD38147, RHD38149-RHD38155, RHD38157-RHD38171, RHD38173-RHD38190, RHD38192, RHD38194-RHD38200, RHD38202-RHD38216, RHD38218, RHD38220-RHD38278.
